# Potential bias of preoperative intravitreal anti-VEGF injection for complications of proliferative diabetic retinopathy

**DOI:** 10.1371/journal.pone.0258415

**Published:** 2021-10-08

**Authors:** Kei Takayama, Hideaki Someya, Hiroshi Yokoyama, Takeshi Kimura, Yoshihiro Takamura, Masakazu Morioka, Hiroto Terasaki, Tetsuo Ueda, Nahoko Ogata, Shigehiko Kitano, Maki Tashiro, Taiji Sakamoto, Masaru Takeuchi

**Affiliations:** 1 Department of Ophthalmology, National Defense Medical College, Tokorozawa, Japan; 2 Department of Ophthalmology, Hyogo College of Medicine, Nishinomiya, Japan; 3 Department of Ophthalmology, University of Fukui Faculty of Medical Sciences, Yoshida, Japan; 4 Department of Ophthalmology, Kagoshima University, Kagoshima, Japan; 5 Department of Ophthalmology, Nara Medical University, Kashihara, Japan; 6 Diabetes Center, Tokyo Women’s Medical University School of Medicine, Tokyo, Japan; Massachusetts Eye & Ear Infirmary, Harvard Medical School, UNITED STATES

## Abstract

**Purpose:**

Intravitreal anti-VEGF injection (IVI) is administered before vitrectomy to assist management of proliferative diabetic retinopathy (PDR)-related complications. In the clinical setting, retinal surgeons determine the use of preoperative IVI based on individual criteria. In this study, we investigated factors related to the potential bias of retinal surgeons in using IVI prior to vitrectomy for PDR-related complications, and evaluated the real-world outcomes of surgeon-determined preoperative IVI.

**Methods:**

Medical records of 409 eyes of 409 patients who underwent 25-gauge vitrectomy for PDR complications at seven Japanese centers (22 surgeons) were retrospectively reviewed. Ocular factors, demographic and general clinical factors, surgical procedures, and postoperative complications were compared between IVI group (patients who received preoperative IVI; 87 eyes, 21.3%) and non-IVI group (patients who did not receive preoperative IVI; 322 eyes, 78.7%). In addition, baseline HbA1c in IVI group and non-IVI group was compared between eyes with and without postoperative complications.

**Results:**

At baseline, IVI group was younger (P<0.001), had shorter duration of diabetes treatment (P = 0.045), and higher frequencies of neovascular glaucoma [NVG] (P<0.001) and tractional retinal detachment [TRD] (P<0.001) compared to non-IVI group. Although IVI group had higher frequencies of intraoperative retinal break and tamponade procedure, there were no significant differences in postoperative complications and additional treatments between two groups. Baseline HbA1c levels were also not correlated with postoperative complications of VH, NVG, and RD both in IVI group and non-IVI group. Logistic regression analysis identified age (P<0.001, odds ratio [OR] 0.95), presence of NVG (P<0.001, OR 20.2), and presence of TRD (P = 0.0014, OR 2.44) as preoperative factors in favor of IVI.

**Conclusions:**

In this multicenter real-world clinical study, younger age and presence of NVG and TRD were identified as potential biases in using IVI before vitrectomy for PDR complications. Eyes that received preoperative IVI had more intraoperative retinal breaks requiring tamponade than eyes not receiving IVI, but postoperative outcome was not different between the two groups.

## Introduction

Proliferative diabetic retinopathy (PDR) features retinal ischemia, neovascularization, and fibrous proliferation, and is one of the most common diseases leading to blindness worldwide [1]. Pars plana vitrectomy (PPV) is generally used for complications of PDR, which include vitreous hemorrhage (VH) and tractional retinal detachment (TRD) [2]. Compared to conventional 20-gauge vitrectomy, PPV using micro-incision vitrectomy surgery (MIVS) decreases surgical invasion, shortens the operating time and duration of hospitalization, and lowers the incidence of intra- and post-operative complications [3,4]. Postoperative outcomes are further improved worldwide by new surgical techniques [5,6] and advanced instruments [7], although postoperative complications may occur in some cases.

Vascular endothelial growth factor (VEGF) is one of the most important factors that promote the development of PDR [8,9]. Since 2007, the use of intravitreal anti-VEGF injection (IVI) has greatly expanded as a treatment for retinal ischemic diseases including PDR [10], and IVI was administered before PPV to assist the management of PDR-related complications [11–13]. Many randomized controlled clinical trials have shown the efficiency and usefulness of preoperative IVI in shortening surgery time [14,15] as well as preventing intraoperative complication [16,17] and postoperative VH [18–21]. Although the merits of IVI in the management of PDR have been reported [22,23], IVI potentially induces TRD by shrinking the proliferative membrane and increasing retinal traction [24–26]. In the clinical setting, retinal surgeons determine the use of preoperative IVI for eyes with PDR-related complications based on individual criteria. In this study, we investigated factors related to the potential bias of retinal surgeons in using IVI before vitrectomy for eyes with PDR complications, and evaluated the real-world outcomes of using preoperative IVI determined by surgeons.

## Methods

### Study design

This study was a multicenter retrospective cohort study conducted at seven centers in Japan. The study adhered to the tenets of the Declaration of Helsinki, and was approved (IRB number: 2725) by the ethics committees of the National Defense Medical College Hospital, University of Fukui Hospital, Hyogo College of Medicine Hospital, Nara Medical University Hospital, Kagoshima University Hospital, Mie University Hospital, and Tokyo Women’s Medical University School of Medicine Diabetes Center. Written informed consent was waived by the ethics committees due to the retrospective nature of the study, but all subjects were informed of the study. The study methods were developed in accordance with the relevant guidelines and regulations, and have been reported previously [27–29]. Some of the data in this report were also used in other studies [27–29].

From the seven tertiary referral centers, the medical records of 409 eyes of 409 consecutive patients (275 males and 134 females) with complications of PDR, who underwent 25-gauge MIVS conducted by 22 retinal surgeons (National Defense Medical College Hospital, 3; Hyogo College of Medicine Hospital, 3; University of Fukui Hospital, 3; Kagoshima University Hospital, 3; Nara Medical University Hospital, 2; Tokyo Women’s Medical University School of Medicine Diabetes Center, 3; and Mie University Hospital, 5) between March 2010 and December 2016 were reviewed retrospectively.

### Study population

Inclusion criteria were: (1) type II diabetic mellitus; (2) the diagnosis of PDR determined by at least 2 retinal surgeons in each center based on preoperative fundus examination, color photographs, intravenous fluorescein angiograms, spectrum domain-optical coherence tomography (SD-OCT), and pre- or intraoperative ultrasonographic findings [30,31]; (3) unresolved VH, TRD, or VH combined with TRD, which were confirmed on B-scan ultrasonography and/or SD-OCT and intraoperative observation; and (4) follow-up period longer than 6 months after the first vitrectomy. Exclusion criteria were (1) past history of other vitreoretinal diseases including retinal vein occlusion, age-related macular degeneration, uveitis, rhegmatogenous retinal detachment, endophthalmitis, proliferative vitreoretinopathy, and trauma; (2) past history of vitrectomy; and (3) follow-up period less than 6 months after the first vitrectomy.

### Preoperative IVI and surgical procedure

The general procedures of preoperative IVI and MIVS in the seven referral centers are described below. IVI given 1–5 days before MIVS by a retinal surgeon was permitted as an optional treatment. For each patient, a 0.05-mL volume (1.25 mg) of bevacizumab was prepared aseptically in a 1.0-mL syringe with a 30-gauge needle, and was injected through the pars plana into the vitreous cavity 3.5 mm posterior to the limbus. After IVI, patients were instructed to apply topical antibiotics.

All patients received peribulbar block under monitored anesthesia care and underwent 25-gauge MIVS using a wide-angle viewing system. Briefly, a surgeon separated the posterior vitreous from the retina by active aspiration using a vitrectomy probe and removed any visible retina-adhering vitreous strands. Intravitreal triamcinolone (40 mg/mL; MaQaid; Wakamoto Pharmaceutical, Tokyo, Japan) was injected into the eye as a marker to facilitate visualization for removal of adherent posterior cortical vitreous strands and additional intraoperative photocoagulation. For all phakic eyes, phacoemulsification and implantation of an artificial intraocular lens using an in-the-bag procedure was performed before the vitrectomy. Surgeons decided whether to perform internal limiting membrane (ILM) peeling using brilliant blue G dye, and tamponade procedures with air, gas, or silicon oil. No eyes received intravitreal or subtenon triamcinolone acetonide injection (STTA) at the end of surgery. Postoperatively, topical antibiotic and anti-inflammatory medications were administered four times/day for a month. During each visit, the patients underwent a complete ophthalmologic examination including measurements of best-corrected visual acuity (BCVA), refractive index, and IOP; slit-lamp and dilated fundus observations (with contact and non-contact examination methods); and SD-OCT. BCVA was measured using a standard Japanese decimal visual acuity chart. We converted the values to logarithm of the minimum angle of resolution (logMAR) scores for data analysis.

### Data collection

Baseline demographic and general clinical data collected were sex, age, diabetes treatment duration, HbA1c, systemic hypertension, anti-coagulation therapy, and estimated glomerular filtration rate. Ocular data collected included baseline data (logMAR, VH, TRD, neovascular glaucoma [NVG] and panretinal photocoagulation [PRP]), use of preoperative IVI, intraoperative retinal break (RB), surgical procedures (ILM peeling, combined cataract surgery, and tamponade [with air, gas or silicon oil]), postoperative complications (VH, NVG, and retinal detachment), and additional postoperative treatments (additional vitrectomy, glaucoma surgery, STTA, and IVI). "Baseline data" was the data at the last examination before surgery, and “postoperative data” were collected for 6 months after the first vitrectomy. NVG was defined as the presence of neovascularization in the anterior chamber angle or iris with an intraocular pressure (IOP) over 21 mmHg. For patients who underwent bilateral vitrectomy, the data of the first operated eye was evaluated.

### Statistical analysis

Shapiro-Wilk test was used to assess normal distribution. Parametric data are expressed as mean ± standard deviation and non-parametric data as median (interquartile range). For continuous data, Mann–Whitey U test was used to compare non-parametric data and Student’s t-test was used to compare parametric data between two groups. Fisher’s test was used to compare categorical data between two groups. Logistic regression analysis was used to detect potential bias in using preoperative IVI. A p level less than 0.05 was considered to be statistically significant.

## Results

### Baseline characteristics

A total of 409 eyes of 409 patients were analyzed. Eighty-seven eyes of 87 patients (21.3%, 55 males and 32 females) received preoperative IVI (IVI group), and 322 eyes of 322 patients (78.7%, 210 males and 112 females) did not receive preoperative IVI (non-IVI group). The baseline characteristics are shown in Table 1.

**Table 1:** General and ocular characteristics at baseline in two groups.

		IVI group (n = 87)	non-IVI group (n = 322)	P
General data				
	Age (year)	50.6±11.9	58.9±12.5	<0.0001^#^
	Sex (male/female)	55/32	210/112	0.80*
	Diabetes treatment duration (year)	10.1±9.1	12.6±10.3	0.045^#^
	HbA1c (%)	7.9±1.9	7.5±1.6	0.074^#^
	Blood sugar (mg/dL)	161.4±63.6	160.9±72.5	0.91^#^
	Estimated glomerular filtration rate (mL/m)	60.7±36.9	59.5±38.3	0.80^#^
	Hypertension	57 (65.5%)	234 (72.7%)	0.19*
	Anti-coagulant use	14 (16.1%)	41 (12.7%)	0.42*
Ocular data				
	Baseline logMAR	1.40 (-0.08–2.70)	1.23 (-0.18–3.0)	0.74^§^
	Neovascular glaucoma	16 (18.4%)	5 (1.6%)	<0.001*
	Vitreous hemorrhage	69 (79.3%)	262 (81.4%)	0.66*
	Tractional retinal detachment	51 (58.6%)	109 (33.9%)	<0.001*
	Panretinal photocoagulation	67 (77.0%)	214 (66.5%)	0.089*

Data are expressed as mean ± standard deviation or number of eyes (%).

^#^Unpaired t-test was used to compare continuous data between two groups.

^§^Mann-Whitney test was used to compare continuous data between two groups.

*Fisher’s test was used to compare categorical data between two groups. IVI group: patients who received preoperative intravitreal anti-VEGF injection, non-IVI group: patients who did not receive preoperative intravitreal anti-VEGF injection.

At baseline, IVI group was significantly younger (50.6 ± 11.9 vs 58.9 ± 12.5, P < 0.001) and had shorter diabetes treatment duration (10.1 ± 9.1 years vs 12.6 ± 10.3 years, P = 0.045) than non-IVI group. Regarding ocular conditions, IVI group had higher frequencies of NVG (18.4% vs 1.6%, P < 0.001) and TRD (58.6% vs 33.9%, P < 0.001) than non-IVI group. Baseline logMAR and frequencies of VH and PRP were not significantly different between two groups.

### Surgical procedures, postoperative complications, and additional treatments

Table 2 shows the surgical procedures in two groups.

**Table 2:** Surgical procedures and intraoperative finding in two groups.

	IVI group (n = 87)	non-IVI group (n = 322)	P
Surgical procedures:			
Cataract surgery	63 (72.4%)	225 (69.9%)	0.65
Internal limiting membrane peeling	35 (40.2%)	126 (39.1%)	0.85
Tamponade procedure	46 (52.9%)	120 (37.3%)	0.0085
Tamponade material: air	23 (26.4%)	76 (24.6%)	0.048
gas	17 (19.5%)	34 (10.6%)	0.005
silicon oil	6 (6.9%)	10 (3.1%)	<0.001
Intraoperative finding:			
Retinal break	35 (40.2%)	68 (21.1%)	<0.001

Data are expressed as number of eyes (%). Fisher’s test was used to compare two groups. IVI group: patients who received preoperative intravitreal anti-VEGF injection, non-IVI group: patients who did not receive preoperative intravitreal anti-VEGF injection.

IVI group had higher frequencies of intraoperative RB (40.2% vs 21.1%, P < 0.001) and requiring tamponade procedure (52.9% vs 37.3%, P = 0.0085) than non-IVI group. In IVI group, air tamponade was used in 23 patients (26.4%), gas in 17 patients (19.5%), and silicon oil in 6 patients (6.9%). In non-IVI group, air, gas and silicone oil were used in 76 patients (24.6%), 34 patients (10.6%) and 10 patients (3.1%), respectively. There were significant differences in the frequency of tamponade materials used between two groups.

Table 3 shows postoperative complications and additional treatments in two groups. Although IVI group had higher frequencies of intraoperative RB and tamponade procedure than non-IVI group, there were no significant differences in postoperative complications (VH, NVG, and retinal detachment [RD]) and additional treatments (additional vitrectomy, glaucoma surgery, STTA, and IVI) between two groups. Since previous study reported a correlation between HbA1c and postoperative complications [32], we also evaluated the correlation of baseline HbA1c with postoperative complications (VH, NVG or RD) in IVI and non-IVI groups. As shown in Table 4, baseline HbA1c levels were not correlated with postoperative complications of VH, NVG, and RD both in IVI group and non-IVI group.

**Table 3:** Postoperative complications and additional treatments in two groups.

		IVI group (n = 87)	non-IVI group (n = 322)	P
Postoperative complications				
	Vitreous hemorrhage	23 (26.4%)	68 (21.1%)	0.29
	Neovascular glaucoma	12 (13.8%)	27 (8.4%)	0.13
	Retinal detachment	4 (4.6%)	10 (3.1%)	0.50
Additional treatments				
	Additional vitrectomy	10 (11.5%)	35 (10.9%)	0.87
	Glaucoma surgery	9 (10.3%)	17 (5.3%)	0.086
	Subtenon triamcinolone injection	3 (3.4%)	7 (2.2%)	0.50
	Intravitreal anti-VEGF injection	4 (4.6%)	16 (5.0%)	0.89

Data are expressed as number of eyes (%). Fisher’s test was used to compare two groups. IVI group: patients who received preoperative intravitreal anti-VEGF injection, non-IVI group: patients who did not receive preoperative intravitreal anti-VEGF injection.

**Table 4:** Comparison of baseline HbA1c in eyes with and those without postoperative complications.

		IVI group		P	non-IVI group		P
		+	-		+	-	
Vitreous hemorrhage							
	Number	23	64		68	254	
	HbA1c	7.7±1.4	8.0±2.1	0.49	7.7±1.6	7.5±1.6	0.55
Neovascular glaucoma							
	Number	12	75		27	295	
	HbA1c	8.6±2.1	7.8±1.9	0.17	7.5±2.0	7.5±1.6	0.99
Retinal detachment							
	Number	4	83		10	312	
	HbA1c	8.9±1.5	7.9±1.9	0.28	7.8±1.3	7.5±1.6	0.68

Data are expressed as mean ± standard deviation or number of eyes. Paired t-test was used to compare two groups. IVI group: patients who received preoperative intravitreal anti-VEGF injection, non-IVI group: patients who did not receive preoperative intravitreal anti-VEGF injection.

### Potential bias factors of using preoperative intravitreal anti-VEGF injections for eyes with PDR

We conducted logistic regression analysis to identify the potential bias factors in using preoperative IVI in eyes with PDR, using baseline general factors (age, sex, and diabetic treatment duration) and baseline ocular factors (baseline logMAR, NVG, TRD, VH, and PRP). The results are presented in Table 5.

**Table 5:** Potential biases of preoperative intravitreal anti-VEGF injection.

Baseline variable factors	P value	Odds ratio	95% confidence interval
Age	<0.001	0.95	0.93–0.98
Sex	0.56	1.18	0.67–2.09
HbA1c	0.67	1.03	0.89–1.20
Diabetic treatment duration	0.29	0.98	0.96–1.01
Baseline logMAR	0.82	0.96	0.68–1.36
Vitreous hemorrhage	0.73	1.13	0.55–2.33
Tractional retinal detachment	0.0014	2.44	1.41–4.22
Neovascular glaucoma	<0.001	20.17	6.36–63.95
Panretinal photocoagulation	0.19	1.51	0.81–2.82

Logistic regression analysis revealed that age (P < 0.001, odds ratio [OR] 0.95, 95% confidential interval [CI]: 0.93–0.98), NVG (P < 0.001, OR 20.17, 95% CI:6.36–63.95), and TRD (P = 0.0014, OR 2.44, 95% CI:1.41–4.22) were potential bias factors in using preoperative IVI.

## Discussion

In the present study, younger age, shorter diabetes treatment duration, higher frequencies of preoperative NVG and TRD, and more intraoperative RB and tamponade procedure were observed in IVI group compared to non-IVI group. Furthermore, younger age and presence of NVG and TRD were identified as potential biases of surgeons in using IVI prior to MIVS for PDR complications.

IVI group was younger and had higher frequencies of preoperative NVG and TRD than non-IVI group. Huang et al. reported that younger PDR patients had significantly higher proportions of active fibrovascular proliferation and TRD, significantly more severe grades, and higher recurrent retinal detachment rate than elder patients [33]. PDR was considered to be more severe in IVI group than in non-IVI group. In fact, intraoperative RB and tamponade procedures were significantly more in IVI group than in non-IVI group. Surgical complications such as recurrence of VH, development of NVG, and retinal detachment are distressing for both surgeons and patients because these could compromise patients’ vision [34]. Higher frequencies of tamponade materials used in IVI group than in non-IVI group may be responsible for the results that there were no significant differences in postoperative complications (VH, NVG, and RD) and additional treatments (additional vitrectomy, STTA, and IVI) between two groups. However, it was also suggested that preoperative IVI reduced postoperative complications.

There were no significant differences in baseline HbA1c and postoperative NVG between IVI group and non-IVI group. We previously reported that young age, shorter diabetes treatment duration, and higher HbA1c were risk factors of developing postoperative NVG [35]. Liang et al. [32] also reported that severe PDR and higher HbA1c were significant prognostic factors of NVG after vitrectomy. Since baseline HbA1c levels were not correlated with postoperative NVG in both groups, whether preoperative IVI benefits patients with high baseline HbA1c is not clear in this study.

Development of PDR in patients with shorter diabetes treatment duration possibly resulted from a longer period of uncontrolled hyperglycemia due to unawareness of the onset of diabetes. On the other hand, since preoperative HbA1c was not significantly different between IVI and non-IVI groups, it is conceivable that urgent surgery was not required in many cases and elective surgery could be performed in most cases after preoperative glycemic control.

In our real-world clinical setting, despite the higher frequencies of preoperative NVG and TRD in patients who received preoperative IVI compared to those who did not, postoperative complications and additional treatments were not different between the two groups. Preoperative IVI has been known to be useful for the management of PDR eyes. Preoperative IVI in PDR patients achieved significantly shorter overall surgery time and smaller number of RB, less intraoperative bleeding, lower incidence of recurrent early postoperative VH, and improved early postoperative visual acuity [18,19], as well as less postoperative retinal detachment and reoperation [36]. Papavasileiou et al. [37] also reported that preoperative IVI reduced the risk of intraoperative complications (RB and VH) and postoperative complications (VH and NVG), resulting in better postoperative anatomic and functional outcomes in PDR eyes. However, except for the report by Papavasileiou et al., the other studies were randomized controlled clinical trials investigating the efficacy of IVI in vitrectomy for complications of PDR. Compared to those reports, the purpose of this study was to identify factors associated with the bias of surgeons in using IVI prior to MIVS for PDR complications, since preoperative IVI was determined by individual surgeons. The difference in baseline characteristics between IVI and non-IVI groups would explain the divergence of our results from those of randomized clinical trials. Although we did not demonstrate improvement in postoperative outcomes as in the study of Papavasileiou et al., our results may suggest that postoperative complications in patients who received IVI might be reduced by preoperative IVI to a comparable level as in patients not requiring IVI.

On the other hand, the timing of preoperative IVI affects postoperative outcome of MIVS for PDR complications [18,19]. Although IVI was performed from 1 to 5 days prior to MIVS in this study, the timing was determined by the surgeons and differed among surgeons and referral centers, and this factor could not be controlled in this study.

The limitations of the present study include the retrospective design, only Japanese participants and surgeons, and not including type 1 diabetic mellitus. Also, preoperative procedures such as the indication and timing of IVI, surgical techniques, and surgery time differed among institutions and surgeons. Furthermore, we did not evaluate preoperative status of vitreous hyaloid or the location and extent of neovascularization. Although all surgical procedures were performed under similar conditions and data collection was relatively complete, some clinicians could have failed to enter all procedural elements.

## Conclusion

This multicenter retrospective study in routine clinical setting revealed the real-world practice and outcomes of PDR treatment with pre-vitrectomy IVI determined by surgeons. Younger age and presence of NVG and TRD were identified as potential biases of surgeons in using IVI before MIVS for PDR complications. Although the frequencies of preoperative NVG and TRD as well as intraoperative RB and tamponade procedure were higher in IVI group than in non-IVI group, there were no significant differences in postoperative complications and additional treatments between the two groups.

## Supporting information

S1 File (XLSX)
